# Which Soft Tissue Sarcoma Patients with Lung Metastases Should not Undergo Pulmonary Resection?

**DOI:** 10.1155/S1357714X02000178

**Published:** 2002-06

**Authors:** Albertus N. van Geel, Joost Rm van Der Sijp, Paul Im Schmitz

**Affiliations:** 1 Department of Surgical Oncology University Hospital Rotterdam/Daniel den Hoed Cancer Groene Hilledijk 301 Rotterdam 3075 EA The Netherlands; 2 Department of Statistics University Hospital Rotterdam/Daniel den Hoed Cancer Rotterdam The Netherlands

## Abstract

Using the second best method of meta-analysis it is significantly shown that patients with an interval of less than 7 months between diagnosis of soft tissue sarcoma and lung surgery for metastases do not benefit.


*Sarcoma* (2002) **6**, 57–60

Taylor&Francis healthsciences

**REVIEW**

### Which soft tissue sarcoma patients with lung metastases should not undergo pulmonary resection?

ALBERTUS N. VAN GEEL[1], JOOST RM VAN DER SIJP[1] & PAUL IM SCHMITZ[2]

*Departments of [1]Surgical Oncology and [2]Statistics, University Hospital Rotterdam/Daniel den Hoed Cancer, Rotterdam, The Netherlands*

**Abstract**

Using the second best method of meta-analysis it is significantly shown that patients with an interval of less than 7 months between diagnosis of soft tissue sarcoma and lung surgery for metastases do not benefit.

### Introduction

Dissemination of soft tissue sarcoma (STS) is in almost all patients restricted to the lungs. For that reason, and due to lack of other effective treatment modalities, a surgical approach of these metastases became more and more popular since the first report of Weinlecher in 1882.[1] After that report, several case reports and unselected small series were published with inconsistent results.

In the last decades several reports have shown a prolonged survival for patients after surgery for lung metastases from STS. In recent series a post-thoracotomy 5-year survival of 30% or more has been found.[2–11] This finding is in contrast with the results from chemotherapy trials with a response rate of 25% or less for a limited period. The question, however, is whether the group of patients with metastasectomy and those with chemotherapy alone are comparable. The patients studied with metastasectomy could be a positive selection with presumed favorable prognostic factors. In fact, the response data for chemotherapy as single treatment for lung metastases and other visceral organs alone are still the subject of EORTC studies and not published yet. (M. van Glabbeke, personal communication) The natural course of STS metastases, if no treatment is given, is not known.[12]

Nevertheless, the general accepted opinion is in contrast to the conclusion of Frost[12] that metastases from STS confirmed in lung are best treated by surgery. In the case of concomitant local recurrence, this recurrence should be treated first in a radical way. Subsequently, the patient will benefit from surgery preferably with low morbidity and a short hospital stay.

One of the old reasons for discussions among thoracic and oncological surgeons is the type of surgical access. For bilateral metastases there is no doubt: a bilateral exploration, sometimes staged, is mandatory.

For unilateral metastases on CT scan, a trend to bilateral approach can be seen, because the number of patients with unsuspected bilateral disease is substantial. Moreover, the morbidity with sternotomy is less than with thoracotomy.[13] The accuracy of the CT scan in predicting the number of metastases was reported to be 50–60%.[14, 15] But with the new generation of CT scanners this figure will increase and the chance of finding unexpected contralateral metastases will decrease dramatically.[16] In close collaboration with the radiologist, all metastases should be studied and the surgeon should decide if radical resection is feasible.

The main goal of this review is to attempt to order the data from the literature and to give guidelines for selection of patients for surgical treatment of lung metastases.

### Prognostic factors

Almost all series report an analysis of factors influencing the prognosis after metastectomy. These factors are age, gender, histology, grade, disease-free interval, number of metastases, and radical resection. The problem is that there is no agreement and a definitive conclusion cannot be made.

The best way to combine results of related studies is to perform a meta-analysis of all raw data from these studies. However, this requires considerable time and effort. Therefore, it is generally accepted to

Correspondence to: A.N.van Geel Md, Phd, Department of Surgical Oncology, University Hospital Rotterdam/Daniel den Hoed Cancer Center, Groene Hilledijk 301, 3075 EA Rotterdam, The Netherlands. Tel: 31 10–4301793; Fax: 31 10–4391011; E-mail: geel@chih.azr.nl

ISSN 1357–714X print/ISSN 1369–1643 online/02/0200057–04 © Taylor & Francis Ltd
DOI: 10.1080/1357714021000022140

use summary data from the published literature, such as hazard rates or expected number of deaths in various subgroups.[17]

Because these summary data are lacking in almost all studies dealing with prognostic factors after surgery of lung metastases from STS we are condemned to the rather crude method of combination of *p* values.[18]

Table 1 shows 10 publications with information about the *p* values of the tests for the influence of disease-free interval and number of metastases on overall survival. It is concluded that the length of the disease-free interval is very important ($p = 0.0002$); But it is not clear which cut-off point must be taken. An interval of at least 7 months or less is a bad prognostic factor. The number of metastases seems not to be an important factor ($p = 0.20$).

### Repeated pulmonary metastasectomy

It is unclear how many patients develop recurrent pulmonary metastases after pulmonary metastasectomy. In fact, only those patients with bilateral thoracotomy can be considered for calculation of the real recurrence rate. In unilateral exploration, occult contralateral metastases can be easily missed as we have shown.[14] About 40% of the patients will relapse after sternotomy, with a median relapse time of about 2 year.[13,15]

The general opinion is that there is no strict contraindication for subsequent pulmonary exploration, either by thoracotomy or sternotomy, although the latter will be more difficult for technical reasons. Up to a number of seven repeated thoracotomies for metastectomy have been reported with long-term survivors. The only restriction is a very short lung relapse time: reoperation within 6 months seems to be useless because these patients will have a bad prognosis anyhow.[19,20]

Like the recent report of Kandioler *et al.*, one has to keep in mind that the data are from series which include all types of malignancies.[21] However, the conclusions drawn from these studies probably are applicable for STS patients as well.

### Video-assisted thoracoscopy

In some centres, pulmonary metastasectomy is performed by video-assisted thoracoscopy (VATS).[22] Although this technique has some advantages to the classical thoracotomy, there is an important reason to question this kind of treatment. Open lung surgery not only offers a good view, but also gives the possibility to palpate the lungs, which is complementary to the preoperative CT staging of the lung metastases. VATS resection should be limited to patients selected by high-resolution CT with only peripheral lung metastases.[23] Tumor implants in the trocart sites have been reported.[24] VATS in combination with a 6–8-cm thoracotomy may be an alternative procedure, because this allows palpation. Also, preoperative CT-guided localization of the metastases may be considered to allow easier resection.[25]

### Future

There is a definitive indication for metastasectomy of lung recurrences of STS if other signs of tumor activity are lacking. The only restriction to be made is that the planned surgery must be radical with curative intent. The general 5-year survival is 25–35%. Surgery alone cannot improve these data; however, bilateral exploration, even in unilateral disease, probably can decrease the number of lung recurrences. But with the greatly improved CT devices (3-mm slices), this conclusion may be criticized.

Reviewing the literature one can see that a lot of patients have had chemotherapy in the course of their metastastic disease; however, never in a consistent way as part of treatment protocol for lung metastases in combination with surgery.[26]

For this reason, some years ago the EORTC Soft Tissue and Bone Sarcoma Group started a prospective trial randomizing between preoperative

**Table 1.** *Analysis of 10 related studies on prognostic factors by combining* p *values*

| Author/year | Number of patients | Interval | *P* | Number of methods | *P* |
|---|---|---|---|---|---|
| Creagan 1979 | 112 | 1 vs >1 year | <0.01 | 1 vs > 1 | NS |
| Takita 1981 | 234 | ≤7 vs >7 months | 0.01 | 1 vs > 1 | <0.01 |
| Jablons 1989 | 55 | ≤1 vs >1 year | 0.03 | <5 vs ≥ 5 | 0.68 |
| Casson 1992 | 58 | ≤1 vs >1 year | 0.90 | ≤ 2 vs >2 | 0.02 |
| Verazin 1992 | 78 | Continuous | 0.02 | ≤5 vs >5 | 0.96 |
| Ueda 1993 | 23 | <6 vs ≥ 6 months | 0.90 | ≤5 vs >5 | NS |
| Saltzman 1993 | 49 | ≤1 vs 1–≤2 vs >2 years | 0.95* | ≤3 vs >3 | 0.85 |
| Robinson 1994 | 44 | ≤1 vs >1 year | NS | ≤2 vs >2 | 0.49 |
| Choong 1995 | 214 | ≤1.5 vs >1.5 year | 0.006 | 1 vs ≥1 | 0.009 |
| Van Geel 1996 | 255 | ≤2.5 vs >2.5 year | 0.003 | ≤3 vs >4 | 0.21 |
| This analysis | | | 0.0002 | | 0.02 |

NS, not significant.

*In text mentioned as significant.

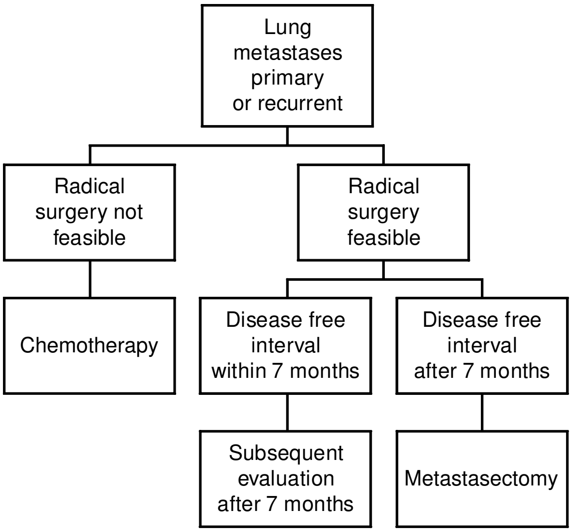

**Fig. 1.** *Algorithm for long metastases from soft tissue sarcoma.*

chemotherapy followed by metastasectomy and metastasectomy alone (EORTC-STBSG 62933). Because the accrual proved to be very difficult, further international collaboration was needed for success. At a certain moment, the study was running, as well as in the EORTC, in the Scandinavian Sarcoma Group (SSG), in the South West Oncology Group (SWOG) and in the Eastern Cooperative Oncology Group (ECOG). Despite these efforts, the study was closed because it was clear that many years were needed for acceptable patient accrual. Therefore, we will never know if the combination of metastasectomy and chemotherapy is better than metastasectomy alone in STS patients. Also, a unique chance to prospectively register patients with lung surgery for STS metastases has been missed.

### Final conclusion

The finding of lung metastases means a dramatically decreased life expectancy for patients with STS. Chemotherapy does not offer cure and, for that reason, surgery is widely accepted as first line treatment in selected patients. The most important selection criterion is the prediction of a radical removal of all visible nodules Other prognostic factors are less important or at least debatable, and grade has been only studied in one series with a strong significance.[11] Using the second best method of meta-analysis, it is significantly shown that patients with an interval of less than 7 months between diagnosis of STS and lung surgery do not benefit. All other parameters, such as age, number of metastases, etc., do not have any influence on prognosis. In fact, the same policy can be followed for recurrent lung metastases (Fig. 1). There is no proof that (neo-) adjuvant chemo-therapy will increase survival in comparison to metastesectomy alone, and is therefore not indicated.

### References

1. Weinlecher J. Wiener Med Wrsch 1882; 20/21.
2. Creagan ET, Fleming TR, Edmonson JH, Pairolero PC. Pulmonary resection for metastatic nonosteogenic sarcoma. *Cancer* 1979; 44: 1908–1912.
3. Takita H, Edgerton F, Karakousis C, Douglas HO, Vincent RG, Beckley. Surgical management of metastases to the lung. *Surg Gynecol Obstet* 1981; 152: 191–4.
4. Jablons D, Steinberg SM, Roth JA, Pittaluga S, Rosenberg SA, Pass HI. Metastasectomy for soft tissue sarcoma. *J Thorac Cardiovasc Surg* 1989; 97: 695–705.
5. Verazin GT, Warneke JA, Dricoll DL, Karakousis C, Ptrelli NJ, Takita H. Resection of lung metastases from soft tissue sarcoma. *Arch Surg* 1992; 127: 1407–11.
6. Casson AG, Putnam JB, Natarajan G, Johnston DA, Mountain C, McMurtey M, Roth JA. Five-year survival after pulmonary metastasectomy for adult soft tissue sarcoma. *Cancer* 1992; 69: 662–8.
7. Ueda T, Uchida A, Kodama K, Doi 0, Nakahara K, Fujii Y, Komatsubara Y, Ono K. Aggressive pulmonary metastasectomy for soft tissue sarcoma. *Cancer* 1993; 72: 1919–25.
8. Saltzman DA, Snyder CL, Ferrell KL, Thompson RC, Leonard AS. Aggressive metastasectomy for pulmonic sarcomatous metastases: a follow-up study. *Am J Surg* 1993; 166: 543–7.
9. Robinson MH, Sheppard M, Moskovic, E. Fisher C. Lung metastasectomy in patients with soft tissue sarcoma. *Br J Radiol* 1994; 67: 129–35.
10. Choong PFM, Pritchard DJ, Rock MG, Sim F Frassica RJ. Survival after pulmonary metastectomy in soft tissue sarcoma. *Acta Orthop Scand* 1995; 66: 561–8.
11. van Geel AN, Pastorino U, Jauch KW, Judson IR, *et al.* Surgical treatment of lung metastases: the EORTC Soft Tissue Bone And Soft Tissue Sarcoma Group study of 255 patients. *Cancer* 1996; 77: 675–82.
12. Frost DB. Pulmonary metastasectomy for soft tissue sarcoma; is it justified? *J Surg Oncol* 1995; 59: 110–5.
13. Pastorino U, Valente M, Gasparini M, Azzarelli A, *et al.* Median sternotomy and multiple lung resections for metastatic sarcomas. *Eur J Cardiothor Surg* 1990; 4: 477–81.
14. van der Veen A, van Geel AN. Median sternotomy; the preferred incision for resection of lung metastases? *Eur J Surg* 1998; 164: 507–12.
15. Pastorino U. *Metastasectomy for soft tissue sarcoma*. In: *Soft tissue sarcoma: present achievements and future prospects*. Boston: Kluwer Academic Publishers, 1997; 65–75.
16. Wright AR, Collie DA. Pulmonary nodules: effect on detection spiral VT pitch. *Radiology* 1999; 3: 837–41.
17. Parmar MKB, Toni V, Steward L. Extracting summary statistics to perform meta-analysis of the published literature for survival endpoints. *Stat Med* 1998; 17: 2815–34.
18. Becker BJ. Combining significance levels. In: Cooper H, Hedges LV, eds. *The handbook of research synthesis*. Ch. 15 (ISBN 0–87154–226–9). New York: Russell Stage Foundation, 1994.
19. van Geel AN, Hoekstra HI, van Coevorden F, Meijer S, *et al.* Repeated resection of recurrent metastatic soft tissue sarcoma. *Eur J Surg Oncol* 1994; 20: 436–40.
20. Progebniak HW, Roth JA, Steinberg SM, Rosenberg SA, Pass HI. Reoperative pulmonary resection in patients with metastatic soft tissue sarcoma. *Ann Thorac Surg* 1991; 52: 197–203.
21. Kandioler D, Kroemer E, Tuechler H, End A, Muller MR, Wolner E, Eckersberger F. Long-term results after repeated surgical removal of pulmonary metastases. *Ann Thorac Surg* 1998; 65: 909–12.

22. Mineo CM, Pompeo B, Ambrogi V, Pistolese C. Video-assisted approach for transxiphoid bilateral lung metastasectorny. *Ann Thorac Surg* 1999; 67: 1808–10.
23. Lin JC, Wiechmann RJ, Szwerc MF, Hazelrigg SR, *et al.* Diagnostic and therapeutic video-assisted thoracic surgery resection of pulmonary metastases. *Surgery* 1999; 126: 636–41.
24. Walsh GL, Nesbitt JC. Tumor implants after thoracoscopic resection of a metastatic sarcoma. *Ann Thorac Surg* 1995; 59: 215–6.
25. RJ Ginsberg. The role of minimally invasive surgery in the diagnosis, staging and treatment of lung cancer. *Lung Cancer*; 29 (Suppl 2): 113 (Abstract).
26. Mentzer SJ, Antman KH, Attinger C, Shemin R, Corson JM, Sugarbaker DJ. Selected benefits of thoracotomy and chemotherapy for sarcoma metastatic to the lung. *J Surg Oncol* 1993; 53: 54–9.
